# Chronic Pruritus in Atopic Patients Treated with Dupilumab: Real Life Response and Related Parameters in 354 Patients

**DOI:** 10.3390/ph15070883

**Published:** 2022-07-17

**Authors:** Luca Mastorino, François Rosset, Federica Gelato, Michela Ortoncelli, Giovanni Cavaliere, Pietro Quaglino, Simone Ribero

**Affiliations:** Dermatologic Clinic, Department of Medical Sciences, University of Turin, Via Cherasco 23, 10121 Turin, Italy; francois.rosset@edu.unito.it (F.R.); federica.gelato@gmail.com (F.G.); mortoncelli@cittadellasalute.to.it (M.O.); gcavaliere@cittadellasalute.to.it (G.C.); pietro.quaglino@unito.it (P.Q.); simone.ribero@unito.it (S.R.)

**Keywords:** pruritus, itching, dupilumab, atopic dermatitis, quality of life

## Abstract

Chronic pruritus is a major symptom of atopic dermatitis (AD). Its etiopathogenesis is complex, and an understanding of the driving factors of its pathogenesis allows for the development of new molecule-targeted therapies. Dupilumab, targeting and blocking interleukin-4 (IL-4) and interleukin-13 (IL-13) molecules, has shown great efficacy in treating AD symptoms such chronic itching. We performed a retrospective observational study to evaluate possible chronic-itch-related characteristics and parameters in 356 AD patients who received dupilumab. The objective of the study was to evaluate the factors associated with the level of pruritus reported by patients at each of the 1575 detections in the form of the peak pruritus numerical rating scale (NRSpp) and sleep disturbance numerical rating scale (NRSsd). We focused on: the eczema area and severity index (EASI), dermatology life quality index (DLQI), patient-oriented eczema measure (POEMS), eosinophilia, L-lactate dehydrogenase (LDH), immunoglobulin E (IgE) and the time from the start of dupilumab therapy. NRSpp fell from 8.6 (sd 1.7) at baseline to 1.7 (sd 2.3) at 36 months and NRSsd from 7 (sd 3) to 0. Regarding the parameters that correlate with NRSpp, all the parameters analysed were significantly correlated except for eosinophils (*p* = 0.136). In the multivariate analysis, both considering and not considering treatment duration, the parameters were correlated (*p* < 0.001); EASI, DLQI, POEM, and LDH significantly correlated with NRSpp (*p* < 0.001 for each, except for LDH *p* = 0.003); while IgE tot lost significance (*p* = 0.337). Similar results were obtained for the parameters correlating with NRSsd. Our results confirm the efficacy of dupilumab on pruritus. The use of questionnaires such as DLQI and POEM is advisable in clinical practice and is adequate for assessing the impact of itching on AD. The low correlation of IgE and eosinophils, the ambiguity of LDH levels with the level of pruritus, and a poor clinical validity and unclear correlation with disease severity suggest a progressive abandonment of monitoring of these values.

## 1. Introduction

Chronic pruritus, as defined as pruritus lasting for more than 6 weeks, is one of the main features of atopic dermatitis (AD) and one of the symptoms that most affects the quality of life (QoL) of atopic patients, causing psychosocial morbidity such as sleep disruption, depression, anxiety and difficulty in concentrating [1]. Its etiology and pathogenesis is complex and is not completely understood [2].

There is no single pathogenetic cause for chronic pruritus: different types of molecular events concerning the interface between skin, keratinocytes and cutaneous nerve fibers are implied. Thus, the lack of comprehension of chronic itch in atopic dermatitis could cause difficulties in managing patients in clinical setting [3].

However, in recent years, the study of this condition and the comprehension of the driving factors in its pathogenesis have allowed for the development of new molecule-targeted therapies that could revolutionize the treatment of chronic itch in patients affected by atopic dermatitis [2,4].

In detail, interleukins (especially the subclasses IL-4 and IL-13) [1] have been shown to play a key role in chronic itch pathophysiology by promoting the initiation and maintenance of a TH2 subset [2], which is the most important cellular subpopulation involved in the development of atopic dermatitis and associated chronic pruritus but not for the development of acute pruritus [5].

The novel monoclonal antibody dupilumab [6] (approved in 2017 by the U.S. Food and Drug Administration for AD), targeting and blocking the IL-4 and IL-13 molecules, has shown great efficacy in treating AD and especially in rapidly improving AD-associated symptoms such as chronic itching [7]. In particular, in a randomized double-blind placebo controlled trial involving adults with moderate-to-severe atopic dermatitis despite treatment with topical glucocorticoids and calcineurin inhibitors, dupilumab in monotherapy resulted in rapid and dose-dependent improvements in clinical indexes in only 4 weeks. The reduction in the pruritus numerical-rating scale score (NRS, ranging from 0 [no itch] to 10 [worst imaginable itch]) at 12 weeks decreased by 55.7%, versus the placebo group where the reduction decreased by only 15.7% (*p* < 0.001) [8]. In another two randomized, placebo-controlled, phase 3 trial of dupilumab versus placebo dupilumab was shown to reduce itching after only 2 weeks, and at 16 weeks it showed an improvement of at least 3 or 4 points in the peak score on the pruritus numerical rating scale in significantly more patients receiving dupilumab than in those receiving a placebo (*p* < 0.001) [9].

All of these findings are strong evidence for supporting the use of dupilumab in the improvement of chronic pruritus related to atopic dermatitis [7].

The biological, clinical, and psychological factors and parameters of AD that correlate with the symptoms of pruritus, particularly during systemic therapy, have not been well-defined yet in the literature on this subject [3]. Real-life experiences of pruritus during treatment with dupilumab and the possible associated factors are scarce, as well as possible differences between adolescent and adult populations [7,10].

## 2. Results

A total of 356 patients were evaluated at baseline, 251(70.5%) at 4 months, 228 (64%) at 8 months, 172 (48.3%) at 12 months, 137 (38.5%) at 16 months, 142 (39.9%) at 20 months, 123 (34.6%) at 24 months, 85 (23.9%) at 28 months, 59 (16.6%) at 32 months, and 22 (6.2%) at 36 months for a total of 1575 visits.

At baseline, 203 patients were male (57%) and 153 were female (43%). The mean age of the population was 39.9 years (sd 17.43), with 21 adolescent patients (<18 yr) and 335 adults (>18 yr). The mean age of onset was 14.4 (sd 20.6), with 219 patients reporting childhood onset (61.5%) and 136 reporting atopic familiarity (38.2%). A total of 19 patients had lesions referable to prurigo excoriate (5.3%), 93 reported predispositions to allergic conjunctivitis (26.1%), 78 reported recurrent herpetic infections (21.9%), and 8 reported previous parasitic infections (2.3%).

For the pruritus scores, NRSpp fell from 8.6 (sd 1.7) at baseline to 1.7 (sd 2.3) at 36 months and NRSsd from 7 (sd 3) to 0. For both scores, a significant decrease was observed as early as 4 months with a subsequent stabilization, and a recrudescence was observed for NRSpp at 24 months (mean 2.4 sd 2.4) and for NRSsd at 20 months (mean 1.9 sd 2.1) (Table 1).

The mean EASI fell from 23.2 at baseline (sd 10.8) to 1.6 (sd 2.02) at 36 months, with a minimum recorded at 20 months (1.25 sd 1.74). Regarding the QoL parameters, DLQI decreased from 14.9 (sd 7) at baseline to 1.2 (sd 2.4) points at 36 months, and POEM decreased from 20.6 (sd 6.1) to 3.8 (sd 5.2) points. For both questionnaires, a partial worsening of values was observed at month 24 (DLQI 3.2 and POEM 5.4).

With regard to laboratory parameters, IgE tot increased from 3239.2 (sd 5020.4) at baseline to 209.3 (sd 308.5) at 36 months, eosinophils from 2.1 (sd 19.6) to 0.38 (sd 0.24), and LDH from 320.9 (sd 144.2) to 198.3 (sd 61.6) (Table 2).

Regarding the parameters that correlate with NRSpp, all the parameters analysed were significantly correlated except for eosinophils (*p* = 0.136, R = 0.04). As EASI increases, the reported itching also increases (*p* < 0.001 R: 0.71, r^2^: 0.5). This behaviour is similar with respect to DLQI (*p* < 0.001 R: 0.76, r^2^: 0.57) and POEMS (*p* < 0.001 R: 0.83 r^2^: 0.69). Regarding the laboratory parameters, IgE positively correlates with NRSpp (*p* < 0.001 R: 0.2, r^2^: 0.04) and LDH levels (*p* < 001, R: 0.33, r^2^: 0.11). The treatment duration negatively correlated with pruritus: as the months of dupilumab therapy increased, NRSpp decreased (*p* < 0.001, R: 0.54, r^2^: 0.29). In the multivariate analysis, both considering and not considering treatment duration,). EASI, DLQI, POEM, and LDH significantly correlated with NRSpp (*p* < 0.001 for each, except for LDH 0.003), while IgE tot lost significance (*p* = 0.337). Following the Pareto diagram for standardized effects, POEM correlated most strongly with NRSpp, followed by DLQI, EASI, months of treatment and LDH (Table 3 and Table 4).

For the parameters correlating with NRSsd, as for the previous score, all the analysed parameters were significantly correlated, as well as the eosinophils (*p* = 0.037, R = 0.06). As EASI increases, so does nocturnal symptomatology (*p* < 0.001 R: 0.71, r^2^: 0.5), and this behaviour is similar with respect to DLQI (*p* < 0.001 R: 0.75, r^2^: 0.5) and POEMS (*p* < 0.001 R: 0.78 r^2^: 0.61). Regarding the laboratory parameters, IgE and LDH positively correlated with NRSsd (*p* < 0.001 R: 0.19, r^2^: 0.04, *p* < 001, R: 0.33, r^2^: 0.11, respectively). Treatment duration negatively correlated with sleep disturbance as the months of dupilumab therapy increased and NRSsd decreased (*p* < 0.001, R: 0.52, r^2^: 0.27). In multivariate analysis, both considering and not considering the duration of treatment (*p* < 0.004), NRSpp, EASI, DLQI, POEM, and LDH significantly with NRSpp (*p* < 0.001 for each, except for LDH 0.003), while IgE tot and eosinophiles lost significance (*p* = 0.337 and *p* = 0.133, respectively). On the Pareto diagram, in this case the correlation with POEM, DLQI and EASI were similar and higher than LDH andmonths of treatment, (Table 5 and Table 6).

**Figure 1:**
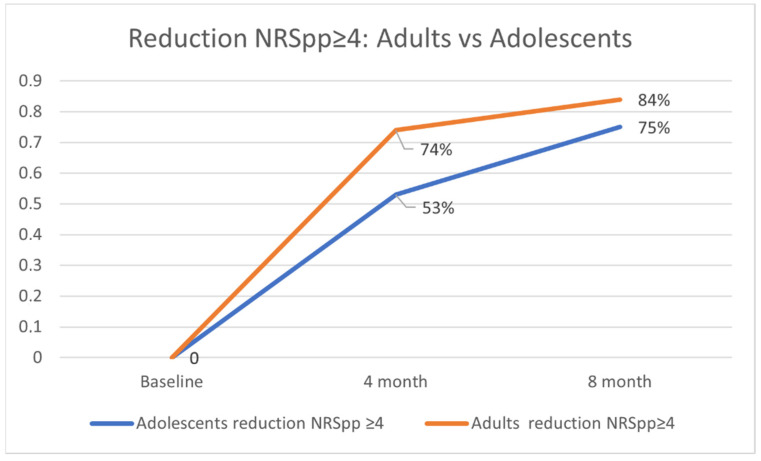
Reduction in NRSpp ≥ 4 adults vs. adolescents.

Regarding the reduction in NRSpp ≥ 4, no significant differences were found between adults and adolescents at 4 and 8 months, although a better performance was observed for the adult population (74% vs. 53% *p* = 0.79 and 84% vs. 75% *p* = 0.529) (Figure 1).

During the 36 months follow-up, ocular adverse events (ocular conjunctivitis) occurred in 44 patients of 352 patients studied (12.53%). In these patients, no interruption of the treatment was necessary since symptoms were successfully managed with topical treatment

## 3. Discussion

The impact of dupilumab on pruritus in adults, adolescents, and children are confirmed by numerous trials and real-life experiences [11,12,13,14,15,16,17]. However, the latter is often limited to low sample sizes and relatively short follow-ups [14,15]. Recently, lebrikizumab and tralokinumab showed good results in the treatment of AD, although results for pruritus control were less promising [18,19]. Nemolizumab is a modern monoclonal antibody specifically synthesised for the management of pruritus, inhibiting IL31, a cytokine widely involved in the pathogenesis of this symptom in acute and chronic AD, and even playing a superior role to IL4 and IL13 [20,21]. Concerning JAK inhibitors, baricitinib, abrocitinib, and upadacitinib are effective in the management of pruritus [22]. In particular upadacitinib was superior to dupilumab in reducing pruritus at 1 and 2 weeks of treatment [23]. The inhibition of JAK ensures the inhibition of receptors; therefore, the subsequent biological effects, from a greater number of cytokines, are variably involved in the pathogenesis of pruritus, not only IL13 and IL4 but also IL31, IL2, IL8, IL25, IL33, IFNγ and thymic stromal lymphoprotein (TSLP) [21,24,25].

Ours is one of the few studies that evaluates the dimension of pruritus in a real-life setting for a total follow-up time of 36 months in a large adult and adolescent population, analysing any differences between the two populations and possible correlations of clinical, laboratory, and psychological factors detectable in clinical practice. These factors are: the extent of disease assessed by EASI; the psychological impact measured by DLQI and POEM (which as we recall are the questionnaires recommended by the Harmonzing Outcome Measures for Eczema (HOME) consensus [26]); and IgEtotals, LDH, and eosinophils, traditionally considered indicators of atopic disease. For the assessment of pruritus, NRSpp and NRSds were used, which are well-defined, reliable, sensitive, and valid scores to assess the intensity of pruritus in the adult population [27]. The value of NRSpp strongly correlates with PROs, such as DLQI and SCORAD, and a reduction of ≥4 is considered an efficacy outcome in both psoriasis and atopic dermatitis [27]. Although NRSpp is widely used in studies on monoclonal antibodies inhibiting IL13 and IL14, it should be remembered that the inhibition of these cytokines results in a reduction in chronic itch and not acute itch; the score analysing only peak itch may overestimate the patient’s itch levels by identifying the acute event by definition [21]. In our population, there was a rapid reduction in all values at month 4, with a progressive reduction until month 36; only the LDH value showed a smaller reduction and more stability throughout the measurements, partially contradicting previous evidence [16]. We did not find substantial differences between the adult and adolescent populations.

When analysing the individual patient visits, we found that neither IgE, nor eosinophils, are associated with the improvement or worsening of itching (both day and night), which may seem surprising but is already well-established in the literature. Eosinophils, if not completely unrelated to the pathophysiology of itching, do not appear to be directly related. Additionally, the role of IgE is more controversial in light of the excellent results achieved by omalizumab in controlling itching in patients with urticaria [25,28]. It is interesting to note that recent studies show a poor correlation between total IgE and eosinophils with the EASI of patients treated with dupilumab, although several studies confirm a reduction in these during dupilumab [16,29]. LDH levels showed a low correlation with the itching reported by our patients [16,30]. There are no studies in the literature correlating LDH with the level of itching; however, in cutaneous lymphoma patients, high LDH levels seem to correlate with more itching in affected patients [31].

POEM, DLQI, and EASI positively correlate with itch levels, regardless of the month of detection. In particular, POEM is more related to daytime itching, it is no coincidence that the HOME consensus selected it as the questionnaire of choice in patients with AD, the questions appear more specific for AD and better intercept the alterations in the quality of life of a DLQI, which remains a generic questionnaire [26]. The role of POEM in the evaluation of night pruritus is similar to DLQI and EASI.

The limitations of our study are those typical of a retrospective observational study. The follow-up, although long in the adult population, is reduced in the adolescent population. Furthermore, the correlations of the various factors were performed on 1557 surveys within a total population of 356 patients, with an average of 4.4 surveys per patient; however, some patients contributed to one survey and others to nine, leading to numerous biases.

## 4. Materials and Methods

### 4.1. Population

We performed a retrospective observational study to evaluate possible pruritus (chronic itch)-related characteristics and parameters in 356 AD patients living in North-West Italy who received dupilumab for the treatment of severe AD from 30 November 2018 to 14 January 2022 at the Dermatology Clinic of the Turin University hospital, a secondary referral centre for the treatment of AD.

The participants included in our study were clinically diagnosed with AD following the criteria proposed by the U.K. Working Party for the diagnosis of AD; in case of doubts, biopsy was performed for histological analysis.

In this prospective cohort study, all the patients treated with dupilumab were included, without any estimation of a sample size.

During this timeframe, 1575 follow-up visits were performed (excluding telemedicine visits caused by COVID-19). All patients received an initial subcutaneous dose of 600 mg dupilumab and, subsequently, 300 mg every other week. Patients were visited at baseline and every 16 weeks. At each visit, CBC and leukocyte formula were assessed, with particular attention toward eosinophils, LDH, and IgE. Patients completed DLQI (dermatology life quality index) and POEMS (patient-oriented eczema measure) before each visit; disease severity was assessed by EASI (eczema area severity index); and pruritus was assessed by NRSpp (numerical rating scale peak of pruritus) and NRSsd (sleep disturbance). At the baseline visit, demographic characteristics such as age, sex, age of onset, presence of nodular prurigo, family history of atopy, predisposition to allergic conjunctivitis and recurrent herpetic recurrences, and history of parasitic infections were recorded.

### 4.2. Objectives

The main objective of the study was to evaluate the factors associated with the level of pruritus reported by patients at each of the 1575 detections in the form of NRSpp and NRSsd. We focused on: EASI, DLQI, POEMS, Eosinophilia, LDH, IgE and time from the start of dupilumab therapy.

The secondary objective was to evaluate the differences in achieving a reduction in NRSpp ≥4 at 4 and 8 months between the adolescent and adult population.

The trend of the analysed parameters was described from baseline to 36 months (T9).

### 4.3. Statistical Analysis

For this analysis, epidemiological data, clinical and QoL-related disease severity were summarized using descriptive statistics. Descriptive statistics were used to evaluate the data set according to the number of patients and their percentage proportion in the groups related to the categorical variables; mean and standard deviation (SD) were used for continuous variables. Inferential statistics were performed up to week 144. To evaluate the association between the analysed parameters and the NRSpp and NRSsd, considering each of the 1575 detections, an initial correlation evaluation was performed through linear regression, which was reported by utilizing the Pearson correlation index R and coefficient of determination r^2^. A multivariate analysis was then performed for significant correlations, using a logistic regression for both NRSpp and NRSsd as dependent variables, and Pareto diagram was used for adjust the weight of each parameter. The categorical variables were analysed using the chi-square test and Fisher’s exact test where needed, while the continuous variables were tested using the Shapiro–Wilk test to investigate the normality of the distribution. Then, the dichotomous normal distributions were compared using a Student’s *t*-test, and non-normal distributions were tested using the Mann–Whitney U test if dichotomous. Kruskal–Wallis tests were used to compare more than 2 distributions. Statistical significance was considered as *p*-value < 0.05.

## 5. Conclusions

Our results confirm the efficacy of dupilumab for itching. The use of questionnaires such as DLQI and POEM in clinical practice is advisable and adequate for assessing the impact of itching on AD. Our data do not reveal a significant correlation between IgE and eosinophils and highlight the ambiguity of LDH levels with regard to the extent of pruritus. The poor clinical validity and unclear correlation with disease severity seem to suggest a progressive abandonment of the periodic monitoring of these values.

## Figures and Tables

**Table 1:** Demographic characteristics.

Demographic Characteristics	
	N°/%
Sex (M)	203 (57%)
Age	39.3 (sd 17.43)
Age of onset	14.4 (sd 20.6)
Adult population	335 (94%)
Childhood onset	219 (61.5%)
Familiarity	136 (38.2%)
Prurigo excoriate	19 (5.3%)
Allergic conjunctivitis	93 (23.1%)
Recurrent herpetic infections	78 (21.9%)
Parasitic infections	8 (2.3%)

**Table 2:** Mean reduction in all parameters analysed.

	Baseline	4 Months	8 Months	12 Months	16 Months	20 Months	24 Months	28 Months	32 Months	36 Months
meanNRSpp	8.6	3.1	2.8	2.7	2.34	1.88	2.4	2.14	1.78	1.73
	sd 1.7	Sd 2.5	Sd 2.3	Sd 2.3	Sd 2.4	Sd 2.1	Sd 2.4	Sd 2.3	Sd 2.2	Sd 2.3
meanNRSsd	7	1	0.6	0.9	0.6	1.9	0.6	0.7	0.3	0
	Sd 3	Sd 2.1	Sd 1.8	Sd 2	Sd 1.5	Sd 2.1	Sd 1.9	Sd 1.8	Sd 1	Sd 0
meanEASI	23.2	3.7	2.8	2.7	2.3	1.3	1.4	1.7	1.5	1.6
	Sd 10.8	Sd 5.3	Sd 3.7	Sd 3.8	Sd 4.1	Sd 1.7	Sd 2	Sd 4	Sd 3.9	Sd 2
meanDLQI	14.9	4.6	4	3.5	2.7	2.4	3.2	2.5	2.6	1.2
	Sd 7	Sd 5	Sd 5	Sd 4	Sd 3.7	Sd 3	Sd 4.2	Sd 3.2	Sd 4.8	Sd 2.4
meanPOEM	20.6	7.3	6.7	6.2	5.7	4.8	5.4	4.9	4.1	3.8
	Sd 6.1	Sd 5.8	Sd 5.9	Sd 5.4	Sd 5.8	Sd 5.4	Sd 5.4	Sd 5.5	Sd 5	Sd 5.2
meanLDH	320.9	236.3	222.4	214.9	201.2	198.9	211.3	203	212.7	198.3
	Sd 144.2	Sd 96	Sd 81.1	Sd 78.7	Sd 70.3	Sd 73.9	Sd 82.6	Sd 59.8	Sd 65.8	Sd 61.6
mean Eosinophiles	2.1	0.64	0.59	0.55	2.07	0.5	1.94	0.35	0.32	0.38
	Sd 19.6	Sd 0.8	Sd 1	Sd 0.9	Sd 17.3	Sd 1.1	Sd 15.3	Sd 0.3	Sd 0.2	Sd 0.2
mean tot-IgE	3239.2	1923	1166	817.8	875.8	551.7	516.9	431.8	379.7	209.3
	Sd 5020.4	Sd 3138.4	Sd 1768	Sd 1220.7	Sd 2366.2	Sd 869.3	Sd 805.7	Sd 728	Sd 634.4	Sd 308.5

**Table 3:** Linear regression for NRSpp.

Linear Regression for NRSpp				
Parameter	Correlation	Pearson R	R^2^	*p*-Value
EASI	0.71	0.71	0.5	<0.001
DLQI	0.76	0.76	0.57	<0.001
POEM	0.83	0.83	0.69	<0.001
Eosinophiles	−0	0	0	0.953
LDH	−0.55	0.55	0.3	<0.001
IgE	0.2	0.2	0.04	<0.001
Months of treatment	−0.54	0.54	0.29	<0.001

**Table 4:** Multivariate analysis for NRSpp with and without months of treatment.

Multivariate for NRSpp					
	Unstandardized Coefficients	Standardized Coefficients			
Model	B	Beta	Standard Error	t	*p*-Value
(Constant)	0.15		0.17	0.87	0.383
LDH	−0	−0.11	0	−5.14	<0.001
IgE tot	−0	−0	0	−0.16	0.875
EASI score	0.08	0.25	0.01	9.49	<0.001
DLQI	0.14	0.29	0.01	9.8	<0.001
POEM	0.12	0.31	0.01	10.13	<0.001
(Constant)	0.15		0.17	0.87	0.383
LDH	−0	−0.11	0	−5.14	<0.001
IgE tot	−0	−0	0	−0.16	0.875
EASI score	0.08	0.25	0.01	9.49	<0.001
DLQI	0.14	0.29	0.01	9.8	<0.001
POEM	0.12	0.31	0.01	10.13	<0.001
Months	−0.03	−0.08	0.01	−4.13	<.001

**Table 5:** Linear regression for NRSsd.

Linear Regression for NRSsd				
Parameter	Correlation	Pearson R	R^2^	*p*-Value
EASI	0.71	0.71	0.5	<0.001
DLQI	0.75	0.75	0.57	<0.001
POEM	0.78	0.78	0.61	<0.001
Eosinophiles	0.03	0.03	0	0.289
LDH	−0.6	0.6	0.36	<0.001
IgE	0.19	0.19	0.04	<0.001
Months of treatment	−0.52	0.52	0.27	<0.001

**Table 6:** Multivariate analysis for NRSsd with and without months of treatment.

Multivariate for NRSsd					
	Unstandardized Coefficients	Standardized Coefficients			
Model	B	Beta	Standard Error	t	*p*-Value
(Constant)	0.15		0.17	0.87	0.383
LDH	−0	−0.11	0	−5.14	<0.001
IgE tot	−0	−0	0	−0.16	0.875
EASI score	0.08	0.25	0.01	9.49	<0.001
DLQI	0.14	0.29	0.01	9.8	<0.001
POEM	0.12	0.31	0.01	10.13	<0.001
(Constant)	0.15		0.17	0.87	0.383
LDH	−0	−0.11	0	−5.14	<0.001
IgE tot	−0	−0	0	−0.16	0.875
EASI score	0.08	0.25	0.01	9.49	<0.001
DLQI	0.14	0.29	0.01	9.8	<0.001
POEM	0.12	0.31	0.01	10.13	<0.001
Months	−0.03	−0.08	0.01	−4.13	<0.001

## Data Availability

Data are available upon reasonable request.

## References

[1] Yosipovitch G., Papoiu A.D.P. (2008). What causes itch in atopic dermatitis?. Curr. Allergy Asthma Rep..

[2] Kido-Nakahara M., Furue M., Ulzii D., Nakahara T. (2017). Itch in Atopic Dermatitis. Immunol. Allergy Clin. North Am..

[3] Mollanazar N.K., Smith P.K., Yosipovitch G. (2016). Mediators of Chronic Pruritus in Atopic Dermatitis: Getting the Itch Out?. Clin. Rev. Allergy Immunol..

[4] Yosipovitch G., Fleischer A.B. (2003). Itch associated with skin disease: Advances in pathophysiology and emerging therapies. Am. J. Clin. Dermatol..

[5] Namer B., Carr R., Johanek L.M., Schmelz M., Handwerker H.O., Ringkamp M. (2008). Separate Peripheral Pathways for Pruritus in Man. J. Neurophysiol..

[6] Seegräber M., Srour J., Walter A., Knop M., Wollenberg A. (2018). Dupilumab for treatment of atopic dermatitis. Expert Rev. Clin. Pharmacol..

[7] Gooderham M.J., Hong HC ho Eshtiaghi P., Papp K.A. (2018). Dupilumab: A review of its use in the treatment of atopic dermatitis. J. Am. Acad. Dermatol..

[8] Thaçi D., Simpson E.L., Beck L.A., Bieber T., Blauvelt A., Papp A., Soong W., Worm M., Szepietowski J.C., Sofen H. (2015). Efficacy and safety of dupilumab in adults with moderate-to-severe atopic dermatitis inadequately controlled by topical treatments: A randomised, placebo-controlled, dose-ranging phase 2b trial. Lancet.

[9] Simpson E.L., Bieber T., Guttman-Yassky E., Beck L.A., Blauvelt A., Cork M.J., Silverberg J.I., Deleuran M., Kataoka Y., Lacour J.-P. (2016). Two Phase 3 Trials of Dupilumab versus Placebo in Atopic Dermatitis. N. Engl. J. Med..

[10] Miniotti M., Lazzarin G., Ortoncelli M., Mastorino L., Ribero S., Leombruni P. (2022). Impact on health-related quality of life and symptoms of anxiety and depression after 32 weeks of Dupilumab treatment for moderate-to-severe atopic dermatitis. Dermatol. Ther..

[11] Silverberg J.I., Yosipovitch G., Simpson E.L., Kim B.S., Wu J.J., Eckert L., Guillemin I., Chen Z., Ardeleanu M., Bansal A. (2020). Dupilumab treatment results in early and sustained improvements in itch in adolescents and adults with moderate to severe atopic dermatitis: Analysis of the randomized phase 3 studies SOLO 1 and SOLO 2, AD ADOL, and CHRONOS. J. Am. Acad. Dermatol..

[12] Simpson E.L., Paller A.S., Siegfried E.C., Boguniewicz M., Sher L., Gooderham M.J., Beck L.A., Guttman-Yassky E., Pariser D., Blauvelt A. (2020). Efficacy and Safety of Dupilumab in Adolescents With Uncontrolled Moderate to Severe Atopic Dermatitis: A Phase 3 Randomized Clinical Trial. JAMA Dermatol..

[13] Paller A.S., Siegfried E.C., Thaçi D., Wollenberg A., Cork M.J., Arkwright P.D., Gooderham M., Beck L.A., Boguniewicz M., Sher L. (2020). Efficacy and safety of dupilumab with concomitant topical corticosteroids in children 6 to 11 years old with severe atopic dermatitis: A randomized, double-blinded, placebo-controlled phase 3 trial. J. Am. Acad. Dermatol..

[14] Mastorino L., Viola R., Panzone M., Avallone G., Gallo G., Ortoncelli M., Cavaliere G., Quaglino P., Ribero S. (2021). Dupilumab induces a rapid decrease of pruritus in adolescents: A pilot real-life study. Dermatol. Ther..

[15] Ribero S., Giura M., Viola R., Ramondetta A., Siliquini N., Cardone P., Tonella L., Quaglino P., Dapavo P., Panzone M. (2020). Effectiveness and safety of dupilumab for the treatment of atopic dermatitis in adult cohort: A real-life Italian tertiary centre experience. J. Eur. Acad. Dermatol. Venereol..

[16] Olesen C., Holm J., Nørreslet L., Serup J., Thomsen S., Agner T. (2019). Treatment of atopic dermatitis with dupilumab: Experience from a tertiary referral centre. J. Eur. Acad. Dermatol. Venereol..

[17] Simpson E.L., Irvine A.D., Eichenfield L.F., Friedlander S.F. (2016). Update on Epidemiology, Diagnosis, and Disease Course of Atopic Dermatitis. Semin. Cutan. Med. Surg..

[18] Guttman-Yassky E., Blauvelt A., Eichenfield L.F., Paller A.S., Armstrong A.W., Drew J., Gopalan R., Simpson E.L. (2020). Efficacy and Safety of Lebrikizumab, a High-Affinity Interleukin 13 Inhibitor, in Adults With Moderate to Severe Atopic Dermatitis: A Phase 2b Randomized Clinical Trial. JAMA Dermatol..

[19] Gutermuth J., Pink A.E., Worm M., Soldbro L., Bjerregård Øland C., Weidinger S. (2022). Tralokinumab plus topical corticosteroids in adults with severe atopic dermatitis and inadequate response to or intolerance of ciclosporin A: A placebo-controlled, randomized, phase III clinical trial (ECZTRA 7). Br. J. Dermatol..

[20] Kabashima K., Matsumura T., Komazaki H., Kawashima M. (2020). Trial of Nemolizumab and Topical Agents for Atopic Dermatitis with Pruritus. N. Engl. J. Med..

[21] Umehara Y., Kiatsurayanon C., Trujillo-Paez J., Chieosilapatham P., Peng G., Yue H., Nguyen H., Song P., Okumura K., Ogawa H. (2021). Intractable Itch in Atopic Dermatitis: Causes and Treatments. Biomedicines.

[22] Silverberg J., Thyssen J., Fahrbach K., Mickle K., Cappelleri J., Romero W., Cameron M., Myers D., Clibborn C., DiBonaventura M. (2021). Comparative efficacy and safety of systemic therapies used in moderate-to-severe atopic dermatitis: A systematic literature review and network meta-analysis. J. Eur. Acad. Dermatol. Venereol..

[23] Blauvelt A., Teixeira H.D., Simpson E.L., Costanzo A., De Bruin-Weller M., Barbarot S., Prajapati V.H., Lio P., Hu X., Wu T. (2021). Efficacy and Safety of Upadacitinib vs Dupilumab in Adults With Moderate-to-Severe Atopic Dermatitis: A Randomized Clinical Trial. JAMA Dermatol..

[24] Garcovich S., Maurelli M., Gisondi P., Peris K., Yosipovitch G., Girolomoni G. (2021). Pruritus as a Distinctive Feature of Type 2 Inflammation. Vaccines.

[25] Buddenkotte J., Steinhoff M. (2010). Pathophysiology and therapy of pruritus in allergic and atopic diseases. Allergy.

[26] Vakharia P.P., Cella D., Silverberg J.I. (2018). Patient-reported outcomes and quality of life measures in atopic dermatitis. Clin. Dermatol..

[27] Yosipovitch G., Reaney M., Mastey V., Eckert L., Abbé A., Nelson L., Clark M., Williams N., Chen Z., Ardeleanu M. (2019). Peak Pruritus Numerical Rating Scale: Psychometric validation and responder definition for assessing itch in moderate-to-severe atopic dermatitis. Br. J. Dermatol..

[28] Barrios D., Phillips G., Geisler A., Trelles S., Markova A., Noor S., Quigley E., Haliasos H., Moy A., Schram A. (2021). IgE blockade with omalizumab reduces pruritus related to immune checkpoint inhibitors and anti-HER2 therapies. Ann. Oncol. Off. J. Eur. Soc. Med. Oncol..

[29] Nettis E., Ferrucci S.M., Pellacani G., Di Leo E., Argenziano G., Foti C., Rongioletti F., Patruno C., Ortoncelli M., Macchia L. (2021). Dupilumab in atopic dermatitis: Predictors of treatment outcome and time to response. J. Eur. Acad. Dermatol. Venereol..

[30] Yamauchi T., Sasaki S., Lee E.S., Tamura T., Seki M., Miwa T., Kobayashi K., Saruta Y., Kitami Y., Sueki H. (2021). Dupilumab treatment ameliorates clinical and hematological symptoms, including blood eosinophilia, in patients with atopic dermatitis. Int. J. Dermatol..

[31] Suga H., Sugaya M., Miyagaki T., Ohmatsu H., Fujita H., Kagami S., Asano Y., Tada Y., Kadono T., Sato S. (2013). Association of nerve growth factor, chemokine (C-C motif) ligands and immunoglobulin E with pruritus in cutaneous T-cell lymphoma. Acta Derm. Venereol..

